# Identifying online racism: Longitudinal pathways to depression symptomology among Black adolescents

**DOI:** 10.1111/jora.70278

**Published:** 2026-10-26

**Authors:** Stephen M. Gibson, Jerica Knox, Fantasy T. Lozada, Brendesha M. Tynes

**Affiliations:** ^1^ Department of Human Development and Quantitative Methodology University of Maryland, College Park College Park USA; ^2^ Department of Psychiatry University of Maryland School of Medicine Baltimore USA; ^3^ Department of Psychology Virginia Commonwealth University Richmond USA; ^4^ USC Rossier School of Education University of Southern California Los Angeles USA

**Keywords:** Black adolescents, depression, online racism, online spaces

## Abstract

Online racism is increasingly becoming a public health concern, with the short‐term psychological consequences of online racism on Black adolescents well documented. Although previous investigations have focused on the unidimensional and cross‐sectional effects of online racism related to depressive symptoms, our understanding of the multidimensional aspects and longitudinal effects of online racism on depressive symptoms among Black adolescents remains limited. Thus, the current investigation contributes to the growing body of literature on online racism exposure among Black adolescents by exploring the longitudinal changes in the identification of online racism exposure and how it is related to later depressive symptoms. Using a sample of 135 self‐identified Black adolescents ranging from 11 to 19 years old (M_age_ = 14 years; SD = 2.44), the results indicated that an increased positive change in individual ORD exposure and vicarious ORD, but not traumatic events online (TEO), was associated with an increase in depression symptoms at wave 2. Overall, these findings highlight the need to consider how changes in Black adolescents' exposure to online racism over time may shape their psychological well‐being and underscore the importance of addressing both direct and indirect forms of online racism as salient developmental stressors for Black adolescents.

## INTRODUCTION

Adolescence is a unique developmental period, marked by identity exploration, heightened sensitivity to social evaluation, and increasing awareness of structural inequalities (Spencer, 2006). During this time, Black adolescents are actively forming their racial worldview and making meaning of their experiences in relation to both peer dynamics and broader sociocultural systems. These normative developmental processes often unfold in the context of exposure to racism and systemic disadvantage, which can increase vulnerability to mental health concerns such as depression (Umaña‐Taylor et al., 2014). In the current online landscape, online spaces and media can foster sociopolitical connection, exploration, and engagement with racial justice movements (Carney, 2016) for Black adolescents; however, it also exposes Black adolescents to high rates of exposure to racism within online spaces, including direct and vicarious attacks, exposure to traumatic racial events, and racialized content embedded in algorithms (Tynes et al., 2018). Online racism has been defined as the subtle or explicit systematic practices of virtual denigration, exclusion, terror, abuse, and dehumanization of groups or individuals based on race that place white individuals at an advantage and people of color at a compounded disadvantage (Tynes et al., 2018). The visibility of police violence, structural racism, and racialized trauma online has only intensified in recent years (Perrin & Atske, 2021; Tynes & Mitchell, 2014), which has been related to increased depressive symptoms among Black adolescents (Keum & Miller, 2017; Tynes et al., 2020; Yip et al., 2019). While there is a breadth of evidence highlighting the effects of online racism on depressive symptoms among Black adolescents (Badejoh et al., 2026; Tynes et al., 2008), there remains a need for longitudinal research to examine how these effects unfold over time (Perrin & Atske, 2021; Tynes & Mitchell, 2014). Additionally, researchers must also consider the positive trajectory of the ability to identify and recognize racism within online racism and the psychological consequences among Black adolescents. The current study addresses this gap by examining how exposure to and recognition of online racism over time relates to subsequent depressive symptoms over a two‐year period among Black adolescents.

### Theoretical grounding

The current study is guided by the Taxonomy of Online Racism Model (Tynes et al., 2018), which offers a comprehensive and multidimensional mapping of the nature and prevalence of online race‐related experiences to which adolescents are exposed. Specifically, the taxonomy model supports systematic investigations into how racism within online environments is maintained, perpetuated, and remains prevalent through bias in online algorithms that shape user experiences, thereby suggesting that online environments are not neutral platforms (Volpe et al., 2021). Additionally, the Taxonomy of Online Racism is especially useful for understanding the various types of online racial stressors that Black adolescents are exposed to. Tynes et al. (2018)'s taxonomy of online racism model argues that online racism can be various acts of digital harm, including online racial discrimination (ORD). ORD is a form of racially targeted harm that takes place in online spaces, includes harmful language, images, or behaviors that are shared through phones, computers, or other electronic devices, and that demean individuals based on their race or ethnicity (Tynes et al., 2020). ORD can manifest in various forms, including individual ORD (i.e., direct attacks, such as being the target of racial insults and threats) and vicarious ORD (i.e., observing others subjected to racial hostility or inclusion).

Moreover, informed by the Taxonomy of Online Racism (Tynes et al., 2018), we recognize that online spaces are influenced by biased algorithms, which reinforce the recurring opportunities that Black adolescents can be exposed to direct and vicarious online racism through online algorithms that perpetuate racist behavior by magnifying an online environment where individuals can actively engage in and practice racist behaviors (Lozada & Tynes, 2017; Keum & Miller, 2018). For example, Everett and Watkins (2008) suggested that biases in automated content moderation resulted in the misrepresentation of marginalized groups, which further suggests that the algorithms of online spaces can be environments where racist stereotypes can be cultivated and implemented. In sum, the Taxonomy of Online Racism Model (Tynes et al., 2018) offers a culturally grounded developmental perspective to interpret how online spaces are exposing Black adolescents to online racialized experiences and subsequent mental harm.

### The online experiences of black adolescents

Online spaces have become integral to adolescent development, serving as key sites for sociopolitical and peer engagement, as well as for developmental processes such as identity exploration and the development of critical consciousness (Giovanelli et al., 2020; Uhis, 2015; Uhis et al., 2017). For Black adolescents, online environments offer opportunities to engage with same‐race peers, access affirming content, and engage in racial socialization messages that may not be available in their immediate contexts. Additionally, previous reports have indicated that Black adolescents spend more time on social media and other online platforms compared to other racial peers (Anderson & Jiang, 2018). Consequently, previous studies have suggested that Black adolescents have reported higher rates of negative online experiences. For instance, Tynes et al. (2015) found that Black adolescents reported exposure to at least one discriminatory incident across three time points. Lozada and Tynes (2017) found that increased engagement with social networking sites, video chat platforms, and online forums was associated with higher rates of occurring and recurring online racism among Black adolescents. Additionally, Tynes et al. (2008) found that Black adolescents reported experiencing both vicarious and individual forms of online racism. This previous work underscores the prevalence of racialized events experienced by Black adolescents within online spaces. Given the prevalence of online racism, Black adolescents' exposure to racist traumatic events online (TEO) has likely spiked over recent years, given the prominent national news coverage regarding structural racism, police brutality, and shootings of Black Americans. This heightened engagement places Black adolescents at greater risk for chronic exposure to racially discriminatory online content that can disrupt developmental processes and contribute to psychological distress. Thus, while online spaces can serve as meaningful spaces for community and identity development, they also may function as racialized settings in which Black adolescents must navigate harmful content, stereotypes, and interpersonal discrimination.

### Mental health consequences of online racial discrimination

In the current sociocultural context, online environments have become a centralized forum to understand how adolescents engage with peers, media, and sociopolitical issues, which makes online spaces a particularly salient site of racialized stress. For Black adolescents, the chronic exposure to racism compounds their vulnerability to depressive symptoms (Lambert et al., 2009; Bernard et al., 2023), which makes the widespread recurring exposure to racist content online particularly harmful. Black adolescents reported increased exposure to pandemic‐related racial stereotypes (Ruiz et al., 2020). Simultaneously, the killings of George Floyd, Breonna Taylor, and others generated viral content documenting racial violence, which many Black adolescents witnessed in real time, and racial intensification within online environments. Furthermore, there is a wealth of evidence underscoring the significant psychological toll, specifically depressive symptoms, on Black adolescents from racialized events (Smith‐Bynum et al., 2014); however, most existing research linking racism and depression has focused on offline (i.e., in‐person) experiences of racial discrimination. There is a growing recognition among scholars to explore additional antecedents of depressive symptoms among Black adolescents, particularly those rooted in online forms of racism. For instance, Keum et al. (2026) found that repeated exposure to negative racialized content online predicted increased depressive symptoms, particularly hopelessness. Additionally, Tynes et al. (2020)'s findings revealed that increased and frequent engagement with online content has been related to higher rates of individual and vicarious forms of online racism among Black adolescents.

While our understanding of the effects of online racism on depressive symptoms among Black adolescents is growing, it still remains incomplete without investigations of the distinct effects of individual and vicarious ORD. For example, Tynes et al. (2008)'s findings revealed that increased exposure to individual ORD was significantly related to an increase in depressive symptoms among Black adolescents; however, they found non‐significant effects when exploring vicarious ORD. Additionally, Tao and Fisher (2022) found that an increase in individual ORD within social media platforms was related to an increase in depressive symptoms among Black adolescents. Thomas et al. (2023) found a similar effect: an increase in individual ORD was associated with more reported depressive symptoms among Black adolescents. Furthermore, one particularly distressing form of online racism is TEO (e.g., exposure to graphic footage or content that depicts racial violence against individuals). Tynes et al. (2019) reported that frequent exposure to TEO was related to higher levels of depressive symptoms. However, with the COVID‐19 pandemic magnifying exposure to negative racialized narratives, especially in online contexts (Stechemesser et al., 2020), limited studies have explored the mental health consequences of this heightened exposure to online racism among Black adolescents (Tynes et al., 2015) in the wake of the COVID‐19 pandemic. Ruiz et al. (2020) indicated that Black adolescents reported increased exposure to TEO linked to pandemic‐related stereotypes and the killings of Black Americans, such as George Floyd. Additionally, much of our existing evidence is based on cross‐sectional data, limiting our understanding of how ORD impacts depressive symptom trajectories over time. Taken together, these findings underscore the urgent need to understand not only how ORD contributes to depressive symptoms longitudinally, but also the psychological consequences of Black adolescents' ability to recognize online racism over time.

### Current study

Black adolescents face both immediate and long‐term consequences of racism (Diemer et al., 2015), and online platforms increasingly expose them to racist content (Hughey & Daniels, 2013). The goal of the current investigation is to explore whether the changes in the recognition of online racism across three dimensions (i.e., individual ORD, vicarious ORD, and TEO) are related to an increase in depression symptoms at a later time point. Finally, we hypothesize that an increased rate of recognition of online racism over time (e.g., wave 1 to wave 2) will be related to higher depressive symptoms among Black adolescents, consistent with prior longitudinal findings linking cumulative racial stress with poor mental health outcomes (English et al., 2020; Tynes et al., 2015).

## METHOD

### Participants

Although 547 self‐identified Black adolescents completed wave 1 of the NCSDL survey, for longitudinal analyses, the analytic sample was restricted to the 135 Black adolescents (ages 11–19; M_age_ = 14.66; SD = 2.44) who completed both waves 1 and 2. Approximately 50% of the sample identified as female. In terms of grade level, 39% were in middle school, 50% were in high school, and 11% had graduated from high school. Participants represented a range of socioeconomic backgrounds: 22% reported a household income of less than $19,999, 14% reported a household income between $20,000 and $39,999, 22% reported a household income between $40,000 and $59,999, 17% reported a household income between $60,000 and $84,999, 10% reported a household income between $85,000 and 99,999, and 16% reported a household income over $100,000. Comparisons between Black adolescents who participated only at wave 1 and those retained at both waves showed no significant differences in age, grade level, income, sex, or study variables.

### Procedure

Data from the current study are from the National Survey of Critical Digital Literacy (NSCDL). The overarching goal of the NSCDL was to conduct the first national, longitudinal, critical‐race mixed‐method study exploring the protective function of critical digital literacy in the association between negative online race‐related experiences and mental health outcomes among racially diverse adolescents. Data were collected using Ipsos Public Affairs, the largest online panel in the U.S. that uses probability‐based sampling methods for recruitment. Survey data collection for wave 1 began in October 2020 and ended in November 2020; for wave 2, it began in October 2021 and ended in November 2021. The NSCDL study was approved by the University of Southern California, Los Angeles' Institutional Review Board (IRB) before data collection, which was first completed in July and August of 2018 for a pilot study and amended for the NSCDL in 2020. Knowledge Panel adhered to all ethical guidelines and followed the research protocol outlined in the IRB application. Data collection procedures consisted of three stages: (1) initial screening for African American and Black households with teens between the ages of 11 and 19 years, (2) distribution of the main survey to eligible respondents, and (3) a follow‐up survey distributed to initial participating respondents 12 months after taking the initial survey. Once screened, participants received an email notification about a new survey available to complete. The median time it took for participants to complete the survey was 14 min.

### Transparency and openness

The protocol of the present study was reviewed and approved by the Institutional Review Board of the University of Southern California, Los Angeles. The data, analytic code, and study materials are not publicly available. This study was not preregistered. The present study is a secondary analysis, and the sample size was determined based on the aims of this larger study.

### Measures

#### Online racial discrimination

The Online Victimization Scale (OVS; Tynes et al., 2010) is a self‐report assessment of adolescents' individual and vicarious victimization experiences across general, sexual, and racial domains. Within the Online Victimization Scale, there are two subscales: Individual Online Racial Discrimination (4 items; “People have threatened me online with violence because of my race or ethnic group.”) using a 3‐point Likert scale (0 = Never, 1 = Once, 2 = More than Once) and Vicarious Online Racial Discrimination (3 items; “People have cracked jokes about people of my race or ethnic group online.”) using a 6‐point Likert scale (0 = Never, 1 = Once, 2 = A few times a year, 3 = A few times a month, 4 = A few times a week, 5 = Every Day). In the current sample, responses across items were averaged to create subscale scores of Individual Online Racial Discrimination (*α* = .74) and Vicarious Online Racial Discrimination (*α* = .75), with both subscales demonstrating adequate inter‐item reliability. Cronbach's alpha statistics were reported by Maxie‐Moreman and Tynes (2022) using a Black sample for Individual Online Racial Discrimination (α = .76).

#### Race‐related traumatic events online

The frequency of participants' experiences of witnessing traumatic race‐related events online (e.g., on Internet news sites, Facebook/Twitter) over the past year was assessed with four self‐report items that corresponded to seeing images or videos of others from their ethnic group being beaten (item 1), arrested or detained (item 2), shot by a police officer (item 3), a viral video of a Black person being shot not by a police officer (item 4). Responses range from 0 (Never), 1 (Once), 2 (A few times a year), 3 (A few times a month), 4 (A few times a week), to 5 (Every Day). In the current sample, responses across items were averaged, with higher scores indicating more frequent exposure to traumatic race‐related events online (*α* = .91). Inter‐item reliability was adequate.

#### Depression

The Children's Depression Inventory (CDI)‐Short, Second Edition (Kovacs, 1985) is a 9‐item self‐report measure of adolescents' depressive symptoms. Participants can choose the statement that best describes them over the past 2 weeks. For example, participants are able to choose between the following statements: (0) None of the statements, (1) I am sad once in a while, (2) I am sad many times, and (3) I am sad all the time. In the current study, responses were averaged across the nine scenarios, with higher scores indicating greater depressive symptoms (*α* = .91). Inter‐item reliability in the current sample was adequate. Symptoms included being sad, crying, feeling alone, and feeling like they had friends. The CDI‐S has shown an internal consistency of .83 with similar samples of Black adolescents (Tynes et al., 2018).

### Data analytic plan

Analyses were conducted using Statistical Package for Social Sciences (SPSS) version 28 (IBM Corp., 2021) and RStudio. An a priori power analysis to determine the necessary sample size for the current investigation. In the present study, 135 participants were sufficient to detect a significant effect (power >0.95, alpha <0.05). Additionally, Little's (1988) Missing Completely At Random test (MCAR test) was conducted to examine the nature of missing data responses. The test results were non‐significant (χ^2^_(14)_ = 15.54, *p* = .342), indicating that the missingness in the dataset is completely random. Given the sample size (*n* = 135) and the percentage of missing values within the variables of interest being less than 2% of the dataset, no further missing data evaluation was conducted. Full information maximum likelihood (FIML) was then applied to handle missing data (Cham et al., 2017). Bivariate correlation analyses were conducted among all study constructs and are displayed in Table 1. Moreover, for the main analyses, we conducted a series of measured variable path analyses (MVPA) using maximum likelihood robust (MLR) estimation to test hypothesized associations between changes in online racism (e.g., difference score of wave 2 and wave 1) across three dimensions (individual ORD, vicarious ORD, and TEO), depressive symptoms at wave 2, and controlling for demographic variables. Model fit was evaluated using multiple indices (χ^2^, RMSEA, SRMR, CFI, TLI), with recommended cutoffs from Hu and Bentler (1999) guiding interpretation. Standardized regression coefficients, standard errors, and significance levels are reported for each model. Demographic variables previously identified as relevant in Black adolescents' online racialized experiences (i.e., biological sex, grade level, media usage; Tynes et al., 2004; Tynes & Mitchell, 2014; Assari et al., 2017) were examined as potential covariates in each model. Independent‐samples *t*‐tests indicated no significant sex differences across most study variables, except that females (*M* = .25, SD = .30) reported significantly higher depressive symptoms at wave 2 than males (*M* = .16, SD = .19). Accordingly, sex was included as a covariate in models predicting wave 2 depression. One‐way ANOVAs revealed no grade‐level differences across study variables; thus, grade was not retained as a covariate.

**TABLE 1:** Correlations, means, and standard deviations for study variables.

		1	2	3	4	5
1.	ΔIndividual online racial discrimination	‐				
2.	ΔVicarious online racial discrimination	.19*	‐			
3.	ΔTraumatic events online	.13	.31**	‐		
4.	Depression W2	.19*	.19*	.06	‐	
5.	Depression W1	.04	.06	−.07	.55**	‐
Means		−.49	−.04	−.15	.21	.27
SD		.69	1.64	1.33	.25	.33

*Note*: *p < .01; **p < .001.

Abbreviation: W, Wave.

## RESULTS

### Preliminary findings

Table 1 presents the bivariate correlations, means, and standard deviations for study variables. On average, Black adolescents reported experiencing depressive symptoms “once in a while” at both wave 1 (*M* = 0.21, SD = 0.25) and wave 2 (*M* = 0.27, SD = 0.33). Participants' reports of online racism experiences varied across type and wave. Individual ORD exposure was infrequent, with adolescents reporting almost never encountering it at both waves (wave 1: *M* = 0.97, SD = 0.74; wave 2: *M* = 0.48, SD = 0.53). In contrast, adolescents reported being exposed to vicarious ORD at least a few times a year for both waves (wave 1: *M* = 2.15, SD = 1.49; wave 2: *M* = 2.12, SD = 1.60). Similar frequencies were observed for TEO, with adolescents reporting exposure a few times per year on average (wave 1: *M* = 2.20, SD = 1.32; wave 2: *M* = 2.06, SD = 1.22). Three change score variables were calculated (wave 2 scores minus wave 1 scores) to model change (Δ) in individual ORD, vicarious ORD, and TEO experiences: ΔIndividual ORD (*M* = −.49, SD = 0.69), ΔVicarious ORD (*M* = −.04, SD = 1.64), and ΔTEO (*M* = −.15, SD = 1.33). Change scores suggest a slight decrease across all online racism dimensions (e.g., individual ORD, vicarious ORD, and TEO) across time. Correlational analyses showed weak to moderate associations among key study variables. Importantly, increases in individual ORD (*r* = .19, *p* < .01) and vicarious ORD (*r* = .19, *p* < .01) were significantly associated with more depressive symptoms, while TEO change was not.

### Measured variable path analysis (MVPA) of ΔOnline racism

#### Model fit

The model fit indices for each model are presented in Table 2. Multiple fit indices were examined prior to model interpretation to ensure the specified parameters were aligned with the variances and covariances. The evaluated parameters are presented in Table 2, including the chi‐square test (χ^2^), Root Mean Square Error of Approximation (RMSEA), Standardized Root Mean Square Residual (SRMR), Comparative Fit Index (CFI), and Tucker–Lewis Index (TLI). Across the models, the χ^2^ test for the hypothesized models was not statistically significant: individual ORD (χ^2^(8) = 15.01, *p* = .059), vicarious ORD (χ^2^(8) = 14.63, *p* = .067), and TEO ORD (χ^2^(8) = 12.93, *p* = .114). While the TLI values were slightly below the recommended .90 cutoff, other indices were within the recommended ranges, indicating an overall adequate model fit (Hu & Bentler, 1999). The following sections present path analyses for each type of online racism, with figures showing standardized regression coefficients, standard errors, and *p*‐values (ΔIndividual ORD – Figure 1; ΔVicarious ORD – Figure 2; ΔTEO – Figure 3).

**TABLE 2:** Fit indices for hypothesized models.

Fit indices	ΔIndividual ORD	ΔVicarious ORD	ΔTraumatic events online
χ^2^	15.01	14.63	12.93
RMSEA	.08	.08	.069
SRMR	.07	.07	.068
TLI	.82	.82	.85
CFI	.90	.90	.91

*Note*: All of the chi‐squared tests (χ^2^) were non‐significant (*p* > .05).

Abbreviations: CFI, comparative fit index; CMIN, chi‐squared to degrees of freedom ratio; ORD, online racial discrimination; RMSEA, root mean squared error of approximation; TEO, Traumatic events online; TLI, Tucker–Lewis index.

**FIGURE 1:**
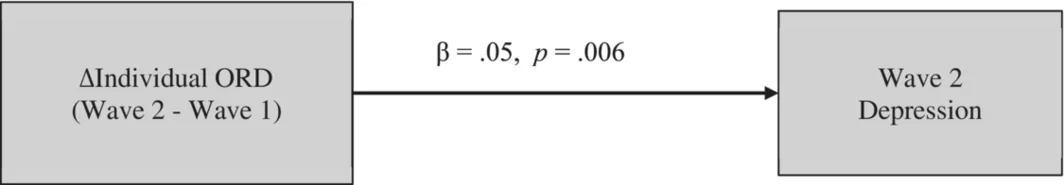
Path diagram and standardized parameter estimates for significant effects in the ΔORD Individual hypothesized models. Non‐significant paths were dashed, and covariates were excluded for simplicity; ORD, Online racial discrimination.

**FIGURE 2:**
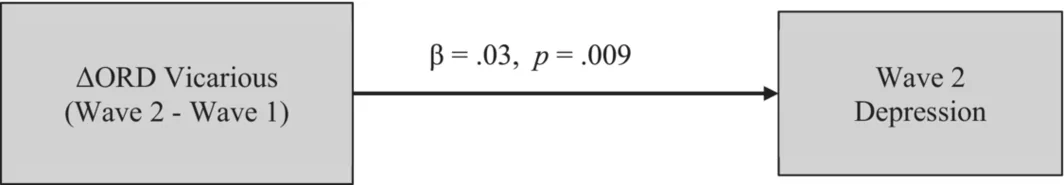
Path diagram and standardized parameter estimates for significant effects in the ΔORD Vicarious hypothesized models. Non‐significant paths were dashed, and covariates were excluded for simplicity; ORD, Online racial discrimination.

**FIGURE 3:**
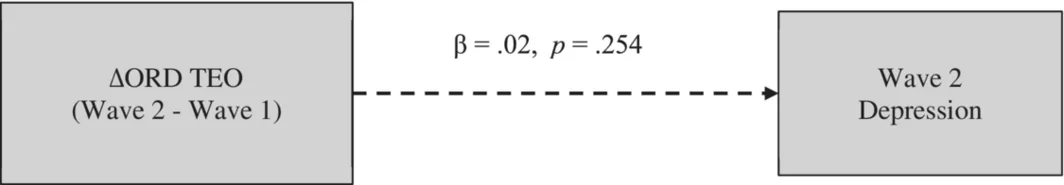
Path diagram and standardized parameter estimates for significant effects in the ΔORD TEO hypothesized models. Non‐significant paths were dashed, and covariates were excluded for simplicity; TEO, Traumatic events online.

#### ΔIndividual ORD

Participants who identified as female (*β* = 0.09, *p* = .005) reported significantly more depressive symptoms at wave two than participants who identified as male. Additionally, participants' depressive symptoms at wave 1 (*β* = 0.46, *p* < .001) were significantly related to reported depressive symptoms at wave 2. Additionally, an increased change in individual ORD experiences (*β* = 0.05, *p* = .006) was positively related to an increase in depressive symptoms at wave 2. The final model explained 36.8% of the variance in depressive symptoms at wave 2.

#### ΔVicarious ORD

Participants who identified as female (*β* = 0.11, *p* = .002) reported significantly more depressive symptoms at wave two than participants who identified as male. Additionally, participants' depressive symptoms at wave 1 (*β* = 0.46, *p* < .001) were significantly related to reported depressive symptoms at wave 2. Additionally, an increased change in vicarious ORD (*β* = .03, *p* = .009) significantly predicted an increase in depressive symptoms at wave 2. The final model explained 37.5% of the variance in depressive symptoms.

#### ΔTraumatic events online

Participants who identified as female (*β* = 0.10, *p* = .007) reported significantly more depressive symptoms at wave two than participants who identified as male. Additionally, participants' depressive symptoms at wave 1 (*β* = 0.48, *p* < .001) were significantly related to reported depressive symptoms at wave 2. However, an increased change in exposure to TEO (*β* = .02, *p* = .254) was not related to depressive symptoms at wave 2. The final model explained 35.3% of the variance in depressive symptoms.

## DISCUSSION

Online spaces are an increasingly salient context for Black adolescents, serving as places where they encounter both positive and negative racial content. Despite growing evidence linking ORD to depressive symptoms (Keum & Miller, 2017; Tynes et al., 2020), most research has been cross‐sectional, offering limited insight into how these effects accumulate over time. The current investigation sought to expand the literature by examining whether the positive changes in recognition of online racism over time were associated with an increase in depressive symptoms among Black adolescents. Grounding our findings in the taxonomy of online racism (Tynes et al., 2018), which highlights how repeated exposure to online racial discrimination across domains, including individual, vicarious, and traumatic events, may accumulate over time, contributing to psychological distress and placing Black adolescents at heightened risk for depressive symptoms. Our findings provide preliminary, longitudinal evidence that increasing exposure to online racism has enduring psychological consequences for Black adolescents. Our findings exploring the effects of positive changes in online racism (i.e., individual ORD, vicarious ORD, and TEO) exposure offer several noteworthy takeaways. First, our results indicated that Black adolescents who reported greater changes in exposure to individual ORD also reported greater depression at the second time point, regardless of their initial levels of depressive symptoms. This finding is supported by a wealth of literature (Maxie‐Moreman & Tynes, 2022; Tao & Fisher, 2022; Tynes et al., 2008) that asserts individual ORD is related to more depressive symptoms among Black adolescents. Clinically, this underscores the need for practitioners to assess exposure to online racism as part of intakes with Black adolescents. Secondly, our findings revealed that greater change in vicarious ORD across time contributes to depressive symptoms among Black adolescents. These findings highlight the importance of measuring the spectrum of online content exposure and offer empirical evidence of the mental health consequences of online vicarious racism among Black adolescents within digital spaces. For instance, Tynes et al. (2008) found that vicarious ORD was not related to psychological adjustment measures (e.g., depression, life satisfaction, and anxiety). Online spaces, specifically social media, provide avenues for exposure to vicarious ORD through racial discourse (e.g., comments, racial jokes, racial stereotypes); however, online spaces also offer an opportunity for Black adolescents to respond and engage in racial discourse, which has been found to provide some protection from online racism (Stewart et al., 2019). Lastly, we found no significant association between reported exposure to traumatic online events over time and subsequent depressive symptoms among Black adolescents. One possible explanation is that online traumatic events, such as viral videos of police brutality, often prompt public commentary and discourse. Such collective engagement may buffer adolescents against internalizing distress, even if the content itself is disturbing (Stewart et al., 2019). This does not suggest that traumatic content is harmless, but rather that the context of viewing, including opportunities to respond, resist, or mobilize, may mitigate its impact. Together, these findings highlight that different forms of online racism exert distinct effects, with individual and vicarious ORD posing particular risks for depressive symptoms.

### Limitations and future research

This study offers several strengths, including a longitudinal design and a multidimensional assessment of online racism within a unique historical context. However, the findings should be interpreted in light of several limitations. First, although the hypothesized model was theoretically grounded, one fit index (TLI) fell below recommended thresholds, raising the possibility of model misspecification. Future research should explore alternative model specifications and conduct sensitivity analyses to strengthen confidence in the pathways tested. Second, measurement limitations must be noted. While this study distinguished between individual ORD, vicarious ORD, and traumatic online events, these constructs may overlap conceptually and empirically. TEO may represent a subset of vicarious ORD, blurring the boundaries between constructs. Future research should employ methods to clarify distinctions across types of online racism. Finally, the current investigation's findings are limited to focusing only on the positive change effects of online racism (i.e., individual ORD, vicarious ORD, and TEO) exposure among Black adolescents; however, future researchers should investigate the protective role of developmental cultural assets (e.g., critical consciousness, ethnic‐racial identity, parental racial socialization, peer support, etc.) on this association between online racism and psychological distress. Additionally, future work should assess not only the presence of racial conversations (e.g., parental racial socialization) but also parents' comfort level and strategies for addressing online racism. Parental interventions should aim to equip parents with tools to discuss digital racism that may help buffer Black adolescents from its psychological toll.

## IMPLICATION

The current investigation's findings indicated that the increased change in online racism has a costly impact on psychological distress among Black adolescents. Furthermore, these findings highlight the long‐term psychological consequences of online racism, and specifically how the identification of more online racism is associated with later depressive symptomatology among Black adolescents. Given that adolescence is a critical developmental stage marked by heightened identity exploration, sensitivity to peer and societal evaluation, and increasing engagement with online environments, our findings underscore the need for coordinated responses across key developmental contexts. For schools, incorporating critical digital literacy into curricula and creating safe classroom spaces where Black adolescents can collectively process online racial events may reduce isolation and stigma while equipping Black adolescents with developmental skills to critically engage with online racial content. Additionally, parental socialization strategies such as the transmission of online and racial messages can play a vital role by affirming positive racial identity and providing coping strategies, while monitoring not just the amount but the quality of Black adolescents' online engagement during this sensitive stage of growth. Lastly, mental health professionals should integrate assessments of online racism exposure into screenings and intakes, support Black adolescents in developing adaptive coping strategies appropriate to their developmental needs, and provide psychoeducation to families and schools about the psychological consequences of online racism. Taken together, these coordinated efforts can validate adolescents' online racial realities, buffer against depressive symptoms, and promote resilience during a developmental period when Black adolescents are especially vulnerable to, but also actively learning to navigate, racialized stressors.

## CONCLUSION

This investigation offers new insights into the long‐term psychological consequences of online racism for Black adolescents. Increases in exposure to individual ORD and vicarious ORD predicted higher depressive symptoms, whereas traumatic online events did not. These findings suggest that Black adolescents' ability to identify and recognize online racism shapes their risk for depressive symptoms, underscoring the importance of considering the role of online algorithms in perpetuating racism within online environments in developmental research. These findings suggest that coordinated efforts from families, schools, and digital platforms are needed to ensure the well‐being of Black adolescents in an era of persistent online racism.

## AUTHOR CONTRIBUTIONS

**Stephen M. Gibson:** Conceptualization; writing – original draft; writing – review and editing; methodology; formal analysis. **Jerica Knox:** Writing – review and editing; conceptualization; formal analysis. **Fantasy T. Lozada:** Supervision; writing – review and editing; conceptualization; methodology. **Brendesha M. Tynes:** Supervision; funding acquisition.

## FUNDING INFORMATION

This research was supported by the Spencer Foundation (Grant Number: 202000140). The funder had no role in study design, data collection, or analysis, the decision to publish, or preparation of the manuscript.

## CONFLICT OF INTEREST STATEMENT

The authors declare no conflicts of interest.

## ETHICAL STATEMENT

This study was approved on March 17, 2020, by the Institutional Review Board of Anonymized's Institutional Review Board (IRB).

## CONSENT

Informed consent/assent was obtained from all participants prior to data collection, in accordance with institutional and APA ethical guidelines.

## Data Availability

The data that support the findings of this study are available from the corresponding author upon reasonable request, subject to institutional/privacy constraints.
